# Contested Medical Knowledge: The College of Physicians’ London *Pharmacopoeia*, Culpeper’s Translations and the Redacted Version of His Publisher Peter Cole

**DOI:** 10.1093/shm/hkag033

**Published:** 2026-07-28

**Authors:** Graeme Tobyn

**Keywords:** Nicholas Culpeper, College of Physicians of London, Pharmacopoeia Londinensis 1618, Pharmacopoeia Londinensis 1650, Peter Cole

## Abstract

England's first pharmacopoeia hid from the untrained knowledge of the medicines physicians prescribed in its Latin text. In the absence of censorship during the interregnum, Nicholas Culpeper (1616–1654) translated it for all to read and appended a commentary pointing out worthless or dangerous remedies to his untrained readers and poor instructions to apothecaries for compounding others. Meanwhile College Fellows were preparing a new updated edition which paid little attention to Culpeper's criticisms and provoked harsher attacks on the College when he translated it in turn. The direction of criticism was reversed when Culpeper's publisher Peter Cole, in anticipation of a new reality in Restoration England, engaged medical editors to rectify and supplement the translation to make it acceptable to the College. This article compares these various texts, highlighting lines of criticism, examples of scurrilous abuse and how the editors defended the official Galenic and chymical remedies of the day.

Nicholas Culpeper (1616–1654) is now well known as an early modern medical educator who translated the leading European medical writers of his time, providing for English doctors a body of work from the best contemporary medical authorities and to his fellow countrymen ‘a whol moddel of physick’ in their native language.^1^ He achieved instant fame, and notoriety, among the fellows of the College of Physicians of London and their supporters, with his first published work of 1649, a translation of the College’s Latin *Pharmacopoeia Londinensis*, first issued in 1618. This was the official list of English medicines which members of the newly created Worshipful Society of Apothecaries were required to make up and dispense, assuming their ‘apothecaries’ Latin’ allowed them to comprehend the formulae, on receipt of a physician’s prescription.^2^ In his *A Physicall Directory: or, A Translation of the Dispensatory Made by the Colledge of Physitians of London* (1649), Culpeper not only rendered into English the lists of simples and the compound preparations made from them for all literate people to read and as an aid to comprehension for the ‘humble retailing apothecary’, but he also added his own explanation of what the simples may be used to treat and critical comments on the origin and authorship, instructions for preparation, safety, usefulness and effectiveness of the book’s compound prescriptions.^3^ These comments were peppered with his mocking and abuse of the College of Physicians for hiding from those unable to afford a physician’s fee a knowledge of medicines inside a Latin text. This study compares and contrasts three editions of this work, produced in a time of great upheaval in English life from the end of the Civil Wars through the Commonwealth Interregnum and into the Restoration period, and explores the factors influencing their variations.

Culpeper’s translation sold very widely, seeing thirty three editions issued up to 1740 while editions of his herbal *The English Physitian* (1652) reached the same number in that period and his *A Directory for Midwives* (1651) managed 31.^4^ It is not surprising that Charles Webster identified Culpeper in his *The Great Instauration* as the prime example of the ideological commitment to the vernacular of the post–Civil War period.^5^

The publication of the pharmacopoeia in English, noted Harold Cook, caused the College much embarrassment.^6^ Supporters of the College immediately responded in print, denigrating the new author in print as a failed apothecary who had run away from his master after lewd debauchery with a whore whom he subsequently married before producing his translation by two years’ drunken labour, ‘mixing every receipt therein with some scruples, at least, of rebellion or atheism, besides the danger of poisoning men’s bodies’, and thus ‘with a handful of conceited knowledge dare[d] challenge all the doctors in the Colledge’.^7^ Culpeper was indeed a rebel, practising astrological medicine illegally in London, seeking the freedom of the individual against a professional class of physicians (not to mention lawyers and priests) intent on maintaining their monopoly over the practice of medicine in London, if not in England, by translating their sacred pharmacopoeia for the public good. His publisher, Peter Cole, who probably proposed to him the idea of rendering the work into English, was, like Culpeper, a sectarian in religion and a republican who knew William Walwyn, the leader of the Levellers, had published illegal pamphlets during the Civil Wars and wrote and issued in 1650 an account of the trial of Charles I for treason and his speech from the scaffold at his execution.^8^

Noting Christopher Hill’s observation that Culpeper was ‘eminently quotable’ and utilising his most resounding phrases to make my points, I have closely compared three editions of Culpeper’s translation: *A Physicall Directory* (1649), his original translation of the December 1618 *Pharmacopoeia Londinensis*, and his *Pharmacopoeia Londinensis; Or, The London Dispensatory, further Adorned by the Studies and Collections of the Fellows now Living of the said Colledg* from 1653 and 1661, both based on the second Latin edition of the *Pharmacopoeia Londinensis* of 1650.^9^ However, the 1661 text was edited by qualified physicians (whom I shall refer to as ‘the medical editors’) already in the employ of, or engaged by the publisher Peter Cole with the result that Culpeper’s scurrilous comments against the College of Physicians of London and other observations perceived to diminish their standing were removed. Table 1 shows the chronological order of publication of these five texts and intermediate editions and how I will title each of them in what follows.

**Table 1.** The *Pharmacopoeia Londinensis* of 1618 and 1650 and Culpeper’s translations of them 1649–1661 with their designed short titles in this study.

Date	Author	Text	Short title
1618	College of Physicians [‘College’]	*Pharmacopoeia Londinensis* 1st Latin edition	1618 Pharmacopoeia
1649	Nicholas Culpeper	*A Physicall Directory: or, A Translation of the Dspensatory made by the Colledge of Physitians of London*. 4to. 1st English edition	A Physicall Directory
		Further editions in 1650 (folio and 2^0^) and 1651 (folio)	Second and third editions of the directory
1650	College of Physicians	*Pharmacopoeia Londinensis* 2nd Latin edition	Second Latin edition
1653	Nicholas Culpeper	*Pharmacopoeia Londinensis: or, The London Dispensatory, further Adorned by the Studies and Collection of the Fellows now Living of the said Colledg* (Folio) 4th English edition	Culpeper’s 1653 translation
		Further editions (5th and 6th) in 1654 (8vo pirated and 8vo and 12mo by P. Cole), 1655, 1656, and 1659 8vo	Later 1650s editions
1661	Nicholas Culpeper and W.R., A.C., J.W. and Abdiah Cole [‘medical editors’]	*Pharmacopoeia Londinensis: or, The London Dispensatory, further Adorned by the Studies and Collection of the Fellows now Living of the said Colledg* Folio. Rational Physicians Library	Cole’s 1661 version

The Latin texts and their translations span very different social contexts. A period of increasing policing of medicine and control over medical publishing under Laudian licencing in the 1630s aided by the establishment of the 1618 Pharmacopoeia had preceded the Civil Wars.^10^ During the Interregnum following the execution of King Charles Culpeper, who had been wounded in the chest defending London for the Parliament, published his various works uncensored and was greeted by success and acclaim. After his death and into the early Restoration period when the full powers of King, Church and State were being re-established, other writers criticised him in print.^11^ The slanderous reference to Culpeper‘s two years’ drunken labour suggests he began translating the pharmacopoeia in 1647. At that time Culpeper claimed to be a busy practitioner.^12^ His training while an apprentice apothecary and his competence in Latin gained at school made him an ideal candidate for the translation.^13^ Culpeper died in 1654 awaiting the Day of Judgement and the return of Christ. After the restoration of Charles II to the throne in 1660 the establishment was wary of any claims to knowledge, including those of popular medicine, that had been treated to radical interpretations by religious sectarians and writers of vernacular medical texts like Culpeper.^14^ Peter Cole was also suspect but maintained his business for a few years until his death in 1665.^15^ Culpeper’s 1653 translation was the last which he produced in his lifetime and represents the fullest commentary on his source material. The 1661 edition is Peter Cole’s extensive revision of that work for changed times, the title-page promising ‘many vertues and uses more than ever were in any former impression’ and was the last version of the pharmacopoeia he would publish. When John Streater purchased the Culpeper titles after Cole’s death, he reissued the pharmacopoeia with most of the redactions of Cole’s 1661 text but missing all the comments from the medical editors, while its preface contained Cole’s designation for its use in Restoration England.^16^

The 1618 Pharmacopoeia had not attracted much research since its comprehensive analysis by George Urdang in 1944 until its treatment in Clare Fowler’s illustrated *Pharmacopoeia Londinensis 1618 and its Descendants* in 2018 issued to celebrate to 400th anniversary of its publication.^17^ Fowler revisited the reasons for publishing a national pharmacopoeia and the muddle over the two editions of 1618 but also included a chapter on Culpeper’s life and work, looking at how his translation *A Physicall Directory* was structured and augmented with additional material and comments so that ‘the contrast between the original *Pharmacopoeia Londinensis* and Culpeper’s version is stark’.^18^ Fowler’s examination of these differences, however, consisted of a brief discussion of the formula for ‘London Treacle’, an example of how simples in the first part of the book were apportioned common English names, Galenic qualities and indications for use, and the explanation of one of Culpeper‘s jokes at the expense of the College in a margin. Among the biographies of Culpeper, only Woolley explored the text of the pharmacopoeia to the extent he described the ingredients of *Confectio Alkermes* and *Mithridate*.^19^ A recent article discussed those compound medicines of the pharmacopoeia which contained hellebore, citing the comments of Culpeper and ‘the medical editors’ from Cole’s 1661 version, but no study has examined all the additions and comments in Culpeper’s several texts before this.^20^

In this article, I explore the development and changing content of these translations: How did Culpeper comment on the text of the 1618 Pharmacopoeia in *A Physicall Directory*, before he was well-known and what had he added or changed by the time of his final version in 1653? What did the medical editors need to excise from the text for Cole’s 1661 version to ensure its appeal to physicians and what can we learn about their stance from their editorial decisions?^21^

Quoting Culpeper at length is bound to threaten what Charles Webster identified as ‘preoccupation with the failure of physicians to fulfil their social responsibilities’—Culpeper‘s main complaint to the College—‘leading observers to neglect the positive aspects of the activities of the College’. These included comparative anatomy, the circulation of the blood and its meaning for physiology and pathology, and the expansion of the materia medica with exotic plant species. The Commonwealth period was thus not a time of intellectual conservatism for College Fellows.^22^ I suggest that, in comparison, revising a predominantly Galenic pharmacopoeia full of old recipes must have seemed limited and tedious to aspiring minds, especially if they lacked experience in the preparation of the compounds. Thus, the contemporary voice of Culpeper had even more to complain about when the second Latin edition emerged and much for the medical editors to remove from Cole‘s 1661 version.

## Culpeper’s *A Physicall Directory* (1649)

Culpeper had in mind from the beginning to make the spare text of the 1618 Pharmacopoeia more usable by the untrained with supplementary material, with a massive expansion of what was only a list of simples in the Latin text by explanations of their medical actions and indications. This also provided a basis on which to surmise the uses of some compound formulae which were unfamiliar to him and, he expected, unknown to many readers. No uses of any simple had been provided in the 1618 Pharmacopoeia, its preface *The Colledge to the Candid Reader* arguing that

Seeing in most dispensaries both ancient and modern the use and virtue of every medicine is described whence ignorant fellows and mountebanks may arm themselves for the practice of physick, and so put a sword into a madman’s hands for the destruction of the Common-wealth, we have added nothing at all of the vertues for we write this to the learned only, and to the nurslings of Apollo, for the health not the understanding of the vulgar.^23^

Culpeper’s catalogue of simples not only included details of Galenic qualities and actions that facilitated their rational use but also explained to the lay reader the difference between herb, root and flower and that roots should be gathered towards the end of summer or at the beginning of spring, and not when the plant had gone to seed, as was commonly thought. He also held and expressed the view that, in general, simples were safer and better than compound medicines. However, around 44% of the 1,190 simples in the pharmacopoeia, some flowers, fruits, seeds, gums and juices were excised where Culpeper considered them unobtainable by his intended readership, because of price or availability (outside of Cheapside or Bucklersbury, the centre of London’s apothecary trade).^24^ Robert Burton had noted that the large range of materials needed to be held in stock by apothecaries to make up the compound remedies of the new pharmacopoeia meant they were often out-of-date and corrupt.^25^

Notwithstanding his apparent amenability in 1649 to these changes, presumably since Culpeper’s necessary enlargement of the section on simples already constituted a significant change from the original, Peter Cole announced the publication of an *exact* translation of the pharmacopoeia in an advertisement in The Parliamentary newsletter *The Moderate: Impartially Communicating Martial Affairs to the Kingdom of England*.^26^ It did not matter, too, that obsequious and out-of-date content had been removed: Theodore de Mayenne’s dedicatory epistle to the long-dead King James never appeared, while the same King’s royal proclamation concerning the pharmacopoeia, the list of the College’s fellows with their titles in 1618 and Culpeper‘s translation of the original address of the ‘Colledge to the candid reader’ only survived in this first translation before being dropped.

For other reasons Culpeper omitted from *A Physicall Directory* the entire section on simple distilled waters, ‘many of which were ridiculous, the simples being not to be obtained green in this land; and sure none in bedlam are so mad as to go about to distil simple water out of dry things’.^27^ He was also inclined to omit the preparation of certain ‘chymical oyls’, since he did not believe the Colledge’s method was the best way to extract them but they were retained. He later would have excised from his 1653 translation the recipes for Avicenna’s Assaireth pills and Mesue’s bdellium pills which the College had left out of their second Latin edition because they were by now ‘seldom used, and therefore are hardly to be had … but the printer will have it put in to stop the mouth of Momus’ and render a faithful translation of the College’s compound medicines.^28^ On weights and measures for simples, Culpeper objected to the inexactness of handfuls and pugils (what ‘you can take up with your thumb and two fingers’) these being ‘as certain as a weather-cock, and as various as men‘s fingers are in length’, not to mention whether the herbs taken were fresh or in dried form.^29^

Culpeper cast the College in his translator’s preface as a ‘a company of proud, insulting, domineering doctors’ who allowed lives to be cast away for want of money to pay them, seeming to echo William Rand’s description of College Fellows as ‘the more ambitious, covetous, domineering and selfish sort of physicians’ in his proposal for a rival College of Graduate Physicians.^30^ Why did they not emulate the Italian physicians, Culpeper enquired, who were paid much less per visit, but earned more by seeing more patients, learned more by attending more cases of sickness and thereby contributed to a commonwealth? Directly addressing the College’s intention of hiding the virtues of the simples and compounds of the pharmacopoeia for fear that the untrained would destroy the commonwealth, with words from the College’s original preface ‘to the candid reader’, Culpeper suggested that if Apollo had served the nine muses as the College served the apothecaries, ‘viz. hid all his art from them, they would have had no more wit than nine geese’. Furthermore, ‘all the nation are already physitians; if you ayl anything, everyone you meet whether man or woman will prescribe you a medicine for it’. Culpeper proposed that anyone with ‘a grain of understanding more than a horse’ could surmise whether his translation would make them more ignorant or more knowing. The apothecaries too would benefit from his translation, he argued, because the compound medicines could not be made up at home by most people, who, learning of their benefits, would seek them from the apothecaries and increase their trade.^31^ Well-to-do ladies who possessed still rooms could perhaps rival apothecaries in the preparation of medicines, but their recipes and wares were prepared not for profit but to serve family, servants and neighbours.

After the commercial and reputational success of the first edition, the directory was issued again in 1650 and a third edition in 1651, each with new prefaces and additional insertions. The 1650 second edition was now titled *A Physical Directory: Or, a Translation of the Dispensatory Made by the Colledge of Physitions of London, and by them Imposed upon All the Apothecaries of England to Make up their Medicines by.* Additions to the text included dosages, especially for those compound medicines containing opium or drastic purgatives, many of which he advised readers to avoid, and a ‘method of ordering the body after sweats and purges’. The risk of endangering people’s lives by making medical knowledge available to the untrained in their own tongue was brought home to Culpeper when feedback from the reception of *A Physicall Directory* required him to make clear in the text that the oils and plasters listed were not for eating but for rubbing into skin or spreading onto cloths and applying to affected parts of the body.^32^ Reports of such misunderstandings led him to call on ‘heaven and earth and all the powers therein to witness, that I was as careful as possibly I could in giving admonitions in every medicine’.^33^

The title page of the third edition of the directory kept the full title of the second edition and now announced the new section *Key to Galen and Hypocrates their Method of Physick* at the back of the book. On safety matters he recalled the view of Hippocrates that physic ‘was never ordained for disorderly and disobedient persons’ and added that its administration was never intended for ‘dunces’.^34^ The preface of this 1651 edition made a mock medical assessment of the ills Culpeper believed the College of Physicians was suffering, the cause of its sickness being Mammon or money, the diagnosis based on how they cheated the rich and neglected the poor, with pride coming before a fall and a haughty mind before destruction prognosticated. The cure involved simples: regard the health of the English people as a king regards his people and, if they were a college of doctors (meaning teachers in Latin), teach them how to preserve their health. ‘As for compounds, take an ounce of honesty, half an ounce of fair dealing, mix them together with a little oil of publick spirit and lay it to your heart’.^35^

## Culpeper’s 1653 translation

The second Latin edition of the pharmacopoeia, published in 1650, has not been subject to close analysis as has the 1618 edition, and here I examine the comments directed at it in Culpeper’s 1653 translation *Pharmacopoeia Londinensis*—these largely featured his own unfavourable comparison with the first Latin edition. Why had the College produced a new version of the pharmacopoeia at that time? Culpeper’s assessment was that the production achieved little except creating the appearance of being busy with an aspect of the health care of the nation, writing concerning a recipe for troches or pastilles of camphor that the College ‘have much altered this, for they must be doing, though to little purpose’. Again, after Cole and Culpeper decided on reinstating those recipes from the 1618 Pharmacopoeia left out in the second Latin edition, Culpeper headed the section on restored linctuses (lohochs) with the words ‘Lohochs left out in their new model, because they must be doing’.^36^

The College was indeed at very low ebb going into 1647 and needed activity to revive it. The college statutes were re-written and, after meetings in July and September 1647, Sir Maurice Williams, Baldwin Hamey and George Ent formed the committee charged with overseeing the production of a new edition of the pharmacopoeia.^37^ Sir George Clark in his history of the College described this new edition as ‘thoroughly and deliberately prepared and entrusted to a new publisher’, fostering a current view that the College wanted to regain publishing rights for the work after Culpeper’s unauthorised translation.^38^ Cole, however, simply made sure at Stationers’ Hall to buy the copyright to the new edition and its English translation from the new publisher Stephen Bowtell to keep control over an English language version.^39^ Clark’s conclusion was that there was ‘little to report which throws light on the attitude of the College to science and thought’ in either this 1650 edition or indeed in the third edition of the pharmacopoeia in 1677, which was almost a reprint of the second Latin edition, with a few medicines added’.^40^

Culpeper was determined to hold the College to the spirit of the Royal Proclamation from James I, (the King being the College’s Jupiter or helping father) which had been inserted into the final version of the 1618 Pharmacopoeia—but not included in the second Latin edition, although the prefatory letter to King James was retained—so that ‘all differences, variations and uncertainties in making or composing of medicines’ were removed and abolished by its issue. As Urdang pointed out, this is the definition of a pharmacopoeia in the modern sense of the word: a pharmaceutical standard intended to secure uniformity in the kind, quality, composition and strength of remedies approved or at least tolerated by the representatives of medicine within a particular political unit and made obligatory for this unit, especially for its pharmacists, by the authorities concerned.^41^ The 1618 Pharmacopoeia was meant to be ‘an anchor firmly cast’ to avoid confusion among apothecaries, so the arrival of a changed second Latin edition had Culpeper complaining that ‘no sooner had I translated their old dispensatory (which should have been authentick till dooms day in the afternoon, had not I done it) to work go they and make another’.^42^ He marvelled at how the old one was cast away like an out-of-date almanac and where slight changes in prescriptions had been inexplicably made—‘here is a grain and a half put in where there was but a grain before’—it was as though such old formulae were now ‘dangerous and destructive to the commonwealth, and so [the physicians] care not a straw for defaming their predecessors, nay some of their own handy works’ in pursuit of their own interests and ‘unconscionable domineering’.^43^ For Culpeper had also noticed that the Fellows had removed the names of the authors of the formulae present in the 1618 original. Culpeper’s slur on the intelligence of College fellows—‘our physitians have but shallow brains, that they are fain to trot as far as Ausberg in Germany to steal receipts’ in the recipe for a linctus of pine nuts; or, about a compound syrup of betony, ‘before the Colledg followed the Augustan physicians to a hair, and indeed who can blame them for following wiser men than themselves? Now they have altered the quantities of the simples’—indicated that he had to hand the Augsberg Pharmacopoeia (*Pharmacopoeia Augustana*) and other sources to compare entries.^44^ Culpeper thought from their ‘apish dispensary’ which disguised the authorship of recipes that either they wanted out of pride for people to think they had devised them themselves, or else, for instance with the *Pulvis antilyssus* which had been titled ‘a powder against the bitings of a mad dog’ in the 1618 Pharmacopoeia or the ointment of sharp-pointed dock which previously was named an ointment for scabs and itch, they dishonestly did not want others to discover their benefits without a prescribing physician.^45^ Culpeper, of course, doubted the ability of College members to make up any such medicines.

Culpeper’s expectation of the stability of a pharmacopoeia complemented his reformist view that tradition was ‘a monster’ and the medical establishment trained young practitioners in the ‘school of tradition and teach them just as a parrot is taught to speak; an author saith so, therefore ‘tis true’.^46^ In practice, however, slight variations of pharmaceutical ingredients posed no difficulty for the learned physician and the skilled apothecary.^47^ Culpeper might have been concerned for the common apothecary and the unlearned lay reader who could be confused by the changing of formulae in updates of his translation of the pharmaceutical text.

Recent studies have documented how in sixteenth-century Italian vernacular ‘descriptive pharmacopoeias’, the ‘tweaking’ of formulae and discussion among apothecaries of their composition and method of making were described.^48^ Such published materials documented some of the tacit knowledge of a profession which had already matured over several centuries of organised medical care in Italian city-states. Tweaking was ‘a low-humming, steady level of activity probing materials, honing methods, and applying small revisions to recipes’ in the local civic antidotaries or official pharmacopoeias involving the ingredients selected, the processes undertaken and the equipment used.^49^ Tweaking thus ‘allowed the apothecary to remain faithful to the inherited tradition of pharmacology, both classical and medieval, while updating and upgrading it to make it viable under local circumstances of production. In doing so, it targeted the recipe’s feasibility, not its reputed effects’.^50^ The tradition of recipes handed down through the ages was thus strengthened: tweaked compound medicines were not, or not considered to be, substantially different to their written formulation. There is no evidence to suggest, however, similar documentation and discussion were taking place among English apothecaries in this period.

Culpeper would have omitted the entire ‘old catalogue of simples’ from his new translation had Peter Cole not insisted it remained, with a promise to issue the edition in smaller print in order not to increase the price of the book for Culpeper’s readership.^51^ Culpeper had had in mind to direct readers to his low-cost herbal, *The English Physitian*, published the year before. Here his readers might learn about native herbs for maintaining their health or curing their diseases ‘and never be beholding to such physitians as the iniquity of these times affords’.^52^ In contrast, the key ingredients of compound medicines in the pharmacopoeia were obtainable only from ‘Greece and Arabia‘, adding to the cost of these ‘outlandish’ herbs which, Culpeper asserted, did not suit English bodies anyway. From a cost point of view, too, Culpeper judged some remedies not worth buying from the apothecary. A tincture of strawberries, new to the 1650 Latin text was ‘a gallant fine thing for gentlemen that have nothing else to do with their money and it will have a lovely look to please their eyes’. Likewise, a julep of Alexandria in the 1618 text, made from rosewater and sugar, was ‘a fine cooling drink in the heat of summer for such as are rich and have nothing else to do with their money’.^53^ Another new recipe in 1650, the *bezoardick powder magisterial* containing sapphires, rubies, jacinths, emeralds, pearls and unicorn horn among 27 ingredients was fit ‘to revive the body, but it will bring the purse into consumption’. Why, Culpeper asked, did the College invent a cordial so expensive that no-one would buy it?^54^

The catalogue of simples in the second Latin edition became the ‘*new* dispensatory’ in Culpeper’s 1653 translation, although he regarded it as ‘the old one new vamped’.^55^ This time he expanded it with a systematic differentiation among the roots, leaves, flowers and other items of the materia medica of their degree of heating, cooling, drying or moistening power, their range of actions on the body and their affinities with parts of the body. This new information supported not only a Galenic allopathic approach to curing but also a Paracelsian one of sympathy/antipathy fitting the style of astrological medicine which was central to the structure of *The English Physitian*.^56^ In his versions of both the old and new catalogues he listed more metals than the College had included, for which he hoped in 1649 ‘my fault is not much’, but by 1653 he no longer cared: ‘whether they were mentioned by the Colledg or no, it matters nothing to me’.^57^

Culpeper was no longer holding back on some topics. Like Galen, he strongly disapproved of the use of *dreckapotheke* or animal excrements but he was not willing to declare his reasons for omitting them from his translation in 1649. Now he rendered into English the section in full in the 1653 ‘old catalogue of simples’ and disdained their use: ‘as for excrements, there the Colledg makes shittin work, and paddle in the turds like Jakes farmers, I will let them alone for fear the more I stir them the more they stink’.^58^ The number of animal parts and excrements, which had nearly quadrupled in number from the May to the December 1618 editions of the pharmacopoeia was augmented again in the 1650 Latin catalogue of simples (excrements increased in number from eleven to thirteen including a differentiation between men‘s and women’s faeces), demonstrating that the College continued to value *dreckapotheke* as simples.^59^ Urdang had been at pains to point out that only pigeon‘s dung was included in one formula for a compound medicine and all other excrements were for external use only.^60^ Culpeper introduced the section with mockery, writing:

Lest any should think I go about to hide from them any thing that might do them good, I have here inserted the living creatures, and excrements, &c., in the order the Colledg left them (for impose them they could not for want of authority; Alack! Alack! The King is dead and the Colledg of Physitians want power to impose the turds upon men).^61^

Culpeper smiled at the thought of which part of the apothecary’s shop the College would appoint such simples to be kept and guessed somewhere near the ‘civit pot’.

Culpeper also translated the new section on simple distilled waters which now differentiated between the simple distilled waters obtained from herbs prepared in common stills set on a good store of ashes, and those requiring digestion in water using tartar, salt or leaven before fractional distillation, any volatile oils being separated off afterwards. Culpeper put in the margin that he knew whom the College had to thank for this way of distillation—presumably a reference to the College’s chemist William Johnson, who had abused Culpeper in print in 1651.^62^ Among the spirits and compound distilled waters, one of the most heavily annotated sections of his translation, Culpeper noted a change to a recipe for angelica water and at first reckoned its purpose to be that more could be charged for the new text with the updated formula in order to keep it away from ‘the vulgars brain’ by being ‘out of the reach of his purse’. Then he realised that the new recipe was the same as a ‘cordial water’ of the 1618 Pharmacopoeia—and that several waters had undergone a name change for the second Latin edition—and asked ‘Pray do but so much as tell what good it doth the vulgar for you to change the names of medicines. I smell a rat, a new trick to cheat the world’.^63^ The ‘vulgar’ were never meant to read the text and anyway medical formulae were bound to be changed, updated and renamed over time. For example, the first century CE Pills of Rufus of Ephesus, translated here by Culpeper (*Pilulae Ruffi*) and formed from the comminution of the three ingredients of aloes, myrrh and saffron mixed with syrup of the juice of lemons to form the pill mass, were still to be found (although by this time minus the saffron) in the British Pharmacopoeia of 1914 as pills of aloes and myrrh with glucose syrup as the excipient.^64^ The same ingredients had been treated alchemically by Paracelsus in the sixteenth century using alcohol distilled from wine and the addition of potassium acetate (’circulatum’) to create the *Elixir Proprietatis.* It was the first elixir to appear in the London Pharmacopoeia, in the third edition of 1677, the taking of which promised to continue health and long life to the utmost limits.^65^

Further additions to the basic text followed, as it had done with every new issue of his translation, in pursuit of providing ‘a whole moddel of physick’ to his fellow countrymen and women, promised in the second edition of the directory.^66^ Substantial introductions appeared to the sections on oils and on compound waters and spirits. To protect his readers from harm by misuse of the powerful distillates, Culpeper warned that they were very heating to the body and were best for those of melancholic and phlegmatic temperaments. He appended his descriptions of the temperaments to help the reader determine whether such medicines were suitable. The fixed oils needed explanations of their actions and uses because the London surgeons whom Culpeper had met and who made most use of oils were not able to affirm them.^67^ Moreover, several oils which continued to be listed in the second Latin edition Culpeper said were no longer in common use. Consequently, readers needed his guidance on these preparations.

Culpeper gave credit where he thought it was due in the second Latin edition, for instance in the College’s improvements of Fernel’s version of a powder of *lithontribon* and Mesue’s formula for a purging apple syrup and in the preparation of a spirit and lesser compound water of wormwood.^68^ Some positive changes in the text he believed were in response to his criticisms of their original formulations in *A Physicall Directory*, for example with the preparation of ‘Imperial water’ where the herbal ingredients were to be infused in rose water and white wine in a glass alembic over hot ashes for 24 hours before distilling.^69^ Whether in hot ashes or hot sand, he warned, ‘all gross bodies stilled in sand will stink egregiously … It seems the Colledge were but mean practitioners of alchymy’; the ingredients needed to be distilled in a water bath instead. All that was written in the 1650 second Latin edition was to distil the ingredients ‘according to art‘. Culpeper commented in his 1653 translation that his earlier observation *‘*so gravelled the Colledg that in their new Dispensatory they quite left out the manner of distillation‘.^70^ Likewise with the recipe for sugar of roses. The 1618 Pharmacopoeia required the dried rose flowers to be reduced to powder in a stone mortar, then boiled with the sugar and rose water, with the option to add four ounces of conserve of red roses to the boiling to give a ‘better colour and the pleasanter tast’.^71^ Culpeper wrote in *A Physicall Directory* not to put the powdered roses in until the sugar and rose water were well boiled, so that the result would keep a red colour and not require the addition of the conserve.^72^ The second Latin edition included this change and Culpeper was able to add in his 1653 translation ‘this they mended as I bid *them’.*^73^

Other changes between the 1618 and 1650 Latin editions offered justifiable reasons for disapproval. With Haly’s powder (*Pulvis Haly*) ‘they have only taken a little pains to less purpose to alter the quantities, else ‘tis the same with their former’, while for the 1650 aromatic powder of roses, ‘they have here left out the musk and ambergreece … for fear the recipe should be too good’. Among the simple oils by expression, almond oil was to be cold pressed in 1618, but ‘in their new moddel’ of 1650 it should be heated in a double vessel and pressed out ‘without heat’, causing Culpeper to exclaim ‘Oh heavens! Did ever the sun shine upon such ridiculous creatures! Who would think a whol Colledg of Physitians should dote so young!’^74^ In another change to a formula, this time for making diacarthamum or purging electuary of safflower, there was not enough moisture in the later version to effect the successful making of an electuary and Culpeper described it as

a pure piece of non-sense. Only let me admire at the pride and headstrongness of the Colledg, that will not mend any error, though as apparent as the sun is up when he is upon the meridian; but *quos perdere vult Jupiter dementis facit*, those whom Jupiter will destroy, he first makes mad; so their Jupiter, or *juvans pater* (as they often in the dedicatory epistle stile the King), and the only God they (poor fooles) worship, unless like heathens they worship many gods, being now sent to take his supper *apud inferos*, hath left such a spirit of madness upon them, which is an absolute forerunner of their end.^75^

Understandably, this reference to the former king supping in hell—Charles, as Culpeper had in mind—never survived the end of the Interregnum, having been excised by the medical editors in Cole’s 1661 version.

Nevertheless, Culpeper was demonstrating his understanding of the provenance, preparation and effects of traditional galenical preparations in the pharmacopoeia and his belief that physicians did not know how to make them up.^76^ The Fellows involved in the production of the 1618 Pharmacopoeia had been supported by ‘some of the most experienced pharmacists [i.e., apothecaries]’ of the day, including John Parkinson, and even if there was active collaboration between physicians and apothecaries over botanical exploration and interest in herbal remedies which assisted a reconciliation between the College and Society of Apothecaries during the Interregnum, this did not amount to physicians being actively involved in pharmacy.^77^ Levitin argued that the flourishing of humanist medicine over the previous century had revived the image of the ancient physician who knew how to prepare the medicines he prescribed and the College physicians acquiring control over apothecaries via the pharmacopoeia was their approximation to this ideal.^78^ Regarding chymical medicines, which historically did not belong to this concept, a proposal to build a chemical laboratory in the gateway to the College at Amen Corner was voted down in 1648 and only in 1651, after the laboratory was established, was William Johnson instructed to prepare certain chemical medicines each month for the inspection and discussion of fellows.^79^

## Cole’s 1661 version

Culpeper’s *Pharmacopoeia Londinensis* (1661) was an expensive folio intended not to be out of place on the bookshelf of a physician and formed part of Cole’s marketing pitch to Restoration readers of medical works he published under the Culpeper brand as the ‘Rational Physicians Library’.^80^ The work was several years in the making, as the mention of the year 1659 by one of Cole’s medical editors shows.^81^ Cole in the preface described his intention to publish an unsurpassable version of the pharmacopoeia in English, issued in folio to match the latest Latin edition of the College page for page and column for column, aiding the comprehension of Latin, the language of science, and the avoidance of dispensing mistakes, and through Culpeper’s explanations of the simples and their uses, to better understand the power and range of the compound medicines.^82^ It would prove useful in supplementing the knowledge of ships’ surgeons who might be required to treat ‘inward diseases’ of shipmates at sea far from a physician, or indeed for ordinary people unable to access a physician close by or when ‘the poverty or low and mean quality of the patients is such that they dare not approach the majesty and splendor of Collegiate, learned and renowned physicians’.^83^ Cole’s sycophancy was intended to attract physicians to a version of their ‘sacred’ book which they had never authorised, in a translated form which revealed what they had tried to keep hidden in the Latin text and so might allow untrained and unlicensed practitioners to employ the same remedies the physicians used, but dangerously, since they were without the knowledge and experience of physicians. Cole himself undoubtedly inserted the following into the body of the text:

These and such like medicaments must be applied after the humours have been prepared, digested, purged, derived, revelled, according to art, and after blood-letting, cupping etc., as occasion shall require and as the art of healing shall suggest, without which art he shall prove but an arrogant and mischievous Cocks-comb, that will go about to administer the medicaments in this book; and which art he may learn if he be not an idle and covetous drone, from the writings of Veslingius, Riolanus, Johnston, Riverius, Sennertus, and *Physick for the Poor* in the English tongue now by God’s providence extant, in which regard ignorant quacksalvers and empiricks shall be justly accountable before God, for their blind and giddy practices, having means to become more understanding, if they had the brains care and conscience to make use thereof. And if they cannot or will not take pains to understand the books aforesaid, are not fit to officiate as Surgeons at sea and in the camp, nor to supply the room of a learned physician in the country or once so much as to look upon a sick person in reference to his cure. And let this warning suffice once for all.^84^

Cole thus trod a fine line between making his English translation of the pharmacopoeia acceptable to physicians and maintaining the wider appeal of his many books on medicine which he continued to publish under the Culpeper name. His business plan no longer implied support for Culpeper’s aim to undermine the College of Physicians’ monopoly on healthcare by providing ‘a whole moddel of physick’ in his works for, as Christopher Hill expressed it, ‘an educated lay public well enough informed to practise a largely preventive medicine and cure simple ailments’ while more difficult medical problems might be tackled free of charge by doctors who were the servants of the community and paid by them.^85^ Rather, Cole looked forward in the Restoration to a return to an orderly status quo in the organisation of medical services to improve the sales of his ‘Rational Physicians’ Library’. To this end, he expressed regret over the tendency of some physicians to compose their own prescriptions instead of ordering medicines described in the pharmacopoeia, which had the consequences of leaving apothecaries with unsold stock and aggravating already challenging relations between the two groups. For the apothecaries’ part, Cole thought they would be more inclined to cease prescribing their own mixtures to those unable to afford or access a physician, for ‘men’s lives ought not to be hazarded through a giddy and affectedly various fashion of prescribing‘, if they were fully employed by physicians and the turnover of their stock proceeded apace and in a timely fashion.^86^ With the restoration of the monarchy, the College increased its efforts to re-establish that monopoly while continuing to serve principally the rich.^87^ Cole, and his nephew Edward Cole who had joined his publishing business, were committed to make as much money as they could from the Culpeper name and arranged to have edited out Culpeper’s ‘unnecessarily’ scurrilous remarks about the English doctors’ love of financial gain above all else so that the dignity of a member of the College would not be tarnished by possession of this new English edition of the pharmacopoeia.

There were other pressing dangers, meanwhile, to Cole and his enterprise in the new Restoration period which required negotiation. When proposals surfaced in July 1661 for legislation that would have given licensing power over medical books to the College of Physicians, Cole who by this time had sunk up to £5,000 into publishing medical books and with his ‘Rational Physitian’s Library’ editions already advertised tried unsuccessfully to obtain a parliamentary exemption in advance.^88^ No notice was taken of his petition, and the following May King Charles II gave assent to the Press Licensing Act, the first of the Restoration period. Fortunately for Cole, this merely repeated the substance of former enactments, among which were the requirement to licence works before their publication, divided into the same classes of books that the 1637 Act had used, but without the draconian punishments for infringement of Archbishop Laud’s day. Instead, while the licencing of medical works such as Cole was producing was part of the duties for which the Archbishop of Canterbury and the Bishop of London took renewed responsibility, their nominees for the task did little more than censor ecclesiastical treatises. The Act effectively became a law concerning printing rather than licencing. Moves against illegal printing and libellous publications ostensibly in the control of the masters and wardens of the Stationers Company, became more effective after August 1663 in the hands of Roger L’Estrange who took over as ‘surveyor of the imprimery and printing presses’ and became the licenser for all printed matter for which no licenser had previously been appointed.^89^ This threatened Cole because L'Estrange, in a tract published earlier that year, had singled out Cole for culpability as a publisher of treasonous and seditious pamphlets during the Interregnum.^90^ This reckoning of the past had perhaps been anticipated at the end of the Interregnum by Culpeper’s widow Alice and his other publisher Nathaniel Brook, who together issued his swansong *Culpeper’s School of Physick* in 1659, containing a biography which is still the main source today of details of his life, and an interpretation of his natal horoscope by John Gadbury along with the last scraps of his notes which had not been published. Cole’s move was to mend fences by removing anything offensive to those in authority from his new edition of the *Pharmacopoeia Londinensis*.

Cole’s 1661 version included new additions to the ‘virtues and uses’ of the compound medicines which Cole had obtained by having ‘earnestly pressed some learned and ingenious gentlemen friends of mine, well known to be both general schollers and able physitians’ to author ‘vertues newly added’ into the text where ‘Mr. Culpeper had wholly omitted them or had spoken more sparingly then the worth of the medicament and thy service did require’.^91^ These medical editors implicitly supported Cole’s reasons for continuing to publish the translation—why not when, for a hundred years of vernacular medical literature in England, there was no simple distinction between lay and professional texts? Indeed, the existing evidence suggests that physicians were among the most avid collectors and readers of medical books in English.^92^ Moreover, they accepted Culpeper’s re-insertions of medicines left out of the revised 1650 edition of the Latin pharmacopoeia. They even chastised the College fellows for leaving out of their 1650 version Mesue’s *confectio humain* among the electuaries, the only remedy in the dispensatory for dimness of sight caused by oppression of the eyes and optic nerve by phlegm and rheum.^93^ In their turn, they dutifully supplied further uses for around 62% of the compound medicines of the pharmacopoeia, based partly on their deep study of ‘the nature of simples and of Man’s constitution’, partly by ‘experience and diligent reading’ (although, wrote Cole, ‘some of my friends bogled at the first’).^94^ on condition that

In this edition of the English dispensatory all the passages reflecting upon the Colledg of London, the authors of the book, should be (for the future) left out. For said they, how can we professing ourselves among the learned endure to see learned men abused out of a capricious humour and in a scurrilous manner? The Colledg is a society of learned men generally and worthy persons; many of them have been our loving friends and acquaintance for many years, and therefore we will not have a hand in the edition of a book that shall use them uncivilly. If divine justice hath chastised that society for some faults of their predecessors best known to themselves…probably now the fault is expiated.^95^

So, who were the medical editors? As the title page records,

In this impression, there is added to the compounds many vertues and uses more than ever were in any former impression. By diverse learned and able doctors of physic, viz. W.R., A.C., J.W. And by Abdiah Cole, doctor of physic, and the liberal arts, who hath practised physick forty nine years, and lived above thirty years out of his own country, and hath seen the practice of France, Italy, Germany, Turkey and the Indies.

It is certain that at least one of them—the doctors’ entries seem to have been written singly rather than as a team, sometimes expressed in the ‘I’ form but never the ‘we’—was actively composing comments in 1659, which matches the publisher Peter Cole’s statement in the opening sentence of his preface that his plan for a more complete edition of the pharmacopoeia had been laid more than two years earlier. Preparations for the new edition were consequently being made towards the end of the Interregnum, possibly initiated by the death of Oliver Cromwell. One editor appears to have been a confirmed bachelor and misogynist who, across the adjacent descriptions of troches of vipers for use in Venice treacle and of Agnus castus, thought the first fit to ‘incite a sleepy husband to dig and manure his parsley-bed and an extravagant whore-haunter to drain his marrow and brain through his conduit pipe’ and the second for those

who (abhorring the insolent usurpation and domineering of wives caused for want of the fitting and just remedy of divorce and allowance of polygamy; and countenanced by the unmanly condescension and dotage of the generality of husbands in this nation and age wherein we live) resolve to enjoy the happy freedom of a single life, without the vexation of carnal desires and danger of scandal which might thence arise.^96^

It is straightforward to identify two of the three medical editors: A.C. is surely Abdiah Cole himself (fl. 1602–1664), the full stop between editors’ initials and Abdiah’s full name perhaps suggesting he was separately the editor-in-chief.^97^ In the book’s preface, Peter Cole may have been referring to Abdiah Cole’s sojourns in the East when he mentioned an editor’s ‘Asiatick and Galenic way of speaking’.^98^ If this is the same Abdiah Cole who as a young Yorkshireman was admitted as a scholar at Trinity College, Cambridge, in 1602, then it is unlikely he was related to Peter Cole, who hailed from Suffolk. Where Abdiah Cole obtained his medical degree is unknown.^99^ Cole was not a member of the College of Physicians. W.R. is almost certain to be William Rowland MD, an unlicensed London medical practitioner from a family of physicians with a foreign medical degree who later claimed responsibility for translating ‘Riverius, Sennertus, Fernelius, Platerus, Rulandus’ among the Culpeper texts issued by Cole after Culpeper’s death. Rowland suffered ‘long captivity in Algier, Tunis and Alexandria’ and was a hostile opponent of astrology, which would not have troubled Peter Cole, who was not the publisher of Culpeper’s astrological works^100^ W.R. was not William Rand MD (1617–1663) who had worked for Peter Cole on translating Jean Riolan’s *Encheiridium Anatomicum et Pathologicum* as *A Sure Guide* (1657) *‘*Englished by Nich. Culpeper, gent. and W.R. doctor of the liberal arts and of physick’. The son of a successful London apothecary, James Rand, who owned a shop on Cheapside where the apothecary shops of London were concentrated, William is best known for a proposal for a ‘College of Graduate Physicians’ among the Hartlib papers, designed to break the monopoly of the College of Physicians. Consequently, he would not have wanted to contribute to a version of Culpeper’s translation which aimed to ingratiate itself with members. By the time of the publication of Cole’s 1661 version, Rand was in poor health and had sunk into obscurity.^101^

It is not known who the remaining physician J.W. is. From the lists of fellows, licentiates and honorary fellows of the College of Physicians in the early Restoration, John Wyberd and James Windet are possible candidates, if the initials really do refer to a College member. Windet may fit the bill in a second way: he was awarded doctor of medicine at Leyden on 26 June 1655, and there are several physicians’ comments in Cole’s 1661 version referring to prescribing habits in Holland, for instance the calling of a linctus a lick-pot ‘from the Hollanders’.^102^ J.W. may otherwise be a Dutch or foreign physician practising in London.

In summary, it may well be that this edition of the pharmacopoeia was indeed produced to appease and appeal to the College of Physicians, but it was not edited and abridged by any of its fellows or members. Nevertheless, the medical editors engaged by Cole seem well connected, one commenting about buckthorn syrup (*syrupus spinae cervine*) ‘I have been informed that the late renowned Sir Theodore Mayerne that old court physician cald this *syrupus domestiens* the housewives syrup intimating the usefulness and harmless nature thereof’.^103^ What these doctors took out, left in, or supplemented of Culpeper’s commentary is exemplified below.

In the 1661 edition, the medical editors excised almost all of Culpeper’s scurrilous comments and attacks on the College of Physicians and replaced them with positive support for the remedies, illustrated by their own examples of good use or additional applications, and sometimes with retorts to Culpeper. Their brief seemingly was to edit and supplement or occasionally entirely replace Culpeper’s comments on the physicians’ text, but not to question the text itself. In this vein they also methodically removed every comment by Culpeper criticising the College’s lists of ingredients and instructions for making up the compound medicines. This sometimes left a rebuttal to Culpeper in an entry without his original offending comment, as if the editors knew that readers would be able to fill in the gaps. For instance, Culpeper objected to one medication of Avicenna being named ‘the ointment of the apostles’ because it contained twelve ingredients:

Here is our Colledges religion *ad unguem* [exactly]. King James is their God, heartsease their trinity, their divinity and holiness in a couple of plaisters, these twelve ingredients are their apostles, their colledg in Amen Corner, where they sing Allelujah, Anglice wood sorrel together.^104^

The editors entirely removed this insinuation of atheism and sought to justify the name of the ointment by stating that Culpeper’s (now no longer evident) quarrelsome remark was impertinent and pointing to biblical references to holy mountains and holy cedars before providing uses for it.^105^ Again, under the recipe for a ‘divine plaster’, Culpeper wondered what the College’s religion was, ‘for they are neither Presbiterians nor independants’.^106^ The editors removed the offending remark and inserted some uses of the plaster ‘wherein much of the wisdom and power and goodness of God is seen in providing such excellent remedies for the accidents that annoy the frail body of Man’.^107^ Similarly, discussing a water of radishes which he reckoned was intended as a purgation for women in childbirth but was ‘a churlish medicine … as fit for it as a sow is for a saddle’, Culpeper did not know if it was the invention of a ‘churlish College’ and took the opportunity to criticise the removal of attribution of authors among the compound remedies of the 1650 text. When in the 1618 Pharmacopoeia ‘they had that ingenuity left to confess where they had their medicines’, he now decided ‘I know not what to say, unless I should say they stole them’.^108^ The editors removed the entry and addressed the qualities of the remedy similarly unknown to them, like Culpeper deducing its applications from its ingredients, being ‘invented (and that with good judgment) for the scurvy, a disease natural to the more Northern parts of our world. Also it moves urine powerfully … and for women that have phlegmatick and impure wombs. Mr Culpeper had not on his considering cap when he past his censure upon this excellent water’.^109^ Again, when Culpeper revealed how the College had hidden the authorship of John of Arderne, the fourteenth-century surgeon, over three simple ointments newly introduced into the second Latin edition, the editors excised the observation then proceeded to name the author and narrate his fame themselves and to add many uses for these ointments and their simples.^110^

The medical editors devoted more time to commenting on some compound medicines more than others. They had little to add to Culpeper’s assessments of syrups, powders and electuaries, although they had to remove and replace entire statements from him on a few of the purging electuaries because of his opposition to the use of drastic cathartics. Conversely, they added to 69% of the entries on the spirits and compound waters and to 82% of the honeys, oxymels and robs, although this may more reflect the prescribing habits or interest of one medical editor dealing with those pharmaceutical forms.^111^ Likewise, 69% of the oils, ointments and plasters for external use were afforded further uses.^112^ One medical editor deconstructed Culpeper‘s method of critique in comments on a receipt for a water and spirit of earthworms. Deemed ‘a mess’ by Culpeper, he declared it to be a private recipe or ‘magistral’ composed by a College fellow or deceased doctor about which the translator ‘was here put to his trumps, not knowing in what book to look for the use and vertues thereof, nor able to find the same in the stars, nor to read in the nature of the ingredients’.^113^ Regarding Culpeper himself, the editors knew of his smoking habit and while agreeing with his fulsome praise of the many therapeutic benefits of tobacco ointment, which they retained, they added ‘however fantastically abused in a riotous manner … ’ tobacco was … ’and therefore cried down by some’. They ventured that if the ‘industrious chymist’ could work his art on it, medicines of great use might be produced.^114^

The exchange created from Culpeper’s original critique of the contents of the pharmacopoeia when subjected to the redactions of the medical editors yielded several themes: the cost, pharmaceutical preparation and safety of the compound medicines, which have already been illustrated, their effectiveness and the new chymical remedies. The medical editors retained Culpeper’s testimonies of effectiveness of a few medicines, some of which he had had to take for his own health. Most significant was his successful use of Mattioli’s bezoar water to treat a hectic fever which might have killed him off before he had translated the pharmacopoeia.^115^ The inclusion of chymical medicines reflected the ‘Elizabethan Compromise’ made by English physicians about Paracelsian preparations by which the remedies were accepted but not the occult philosophy which explained them.^116^ Culpeper seemingly accepted both in his system of astrological medicine: and on medicines at least, he approved of distillations of herbs but seemed agnostic on truly ‘chymical preparations’. When he commented on the antimonial wines among the compounds he did so for reasons of safety, not concerning substance. He was very cautious about having his inexperienced readers involve themselves with medicines causing vomiting and he discussed together the three antimonial vomits among the physical wines, writing:

vomits are fitting medicines but for a few…the mouth being ordained to take in nourishment, not to cast out excrements and to regulate a man’s body in vomiting; and the doses of vomits require a deeper study in physick than I doubt the generality of people yet have. I omit it therefore at this time…because I would not willingly have them do themselves a mischief.^117^

Since Culpeper had not entered separate comments for the first two wines, the vomiting claret and the *vinum benedictum*, the medical editors had to provide entries instead (’vertues newly added‘), which concerned their degrees of ‘churlishness’ or difficulty in stomaching and their dosages. However, his comment above under the third wine, on the dangers of dosing by the untrained, they allowed to remain, and they wrote nearly a whole column of text on the virtues of this third antimonial wine made with regulus of antimony, which they considered equivalent to the antimonial cup. The dose was ‘about an ounce, little more or less … it is safe and without danger and never loseth nor diminisheth its vertues’, they affirmed.^118^

Across these themes relating to the contents of the *Pharmacopoeia Londinensis* Culpeper took up the role of translator-practitioner, as defined by Alberts, Fransen and Leong in their summary of case studies of translations of medical texts, by virtue of his expertise, in Latin and in pharmacy after six years (1634–40) in apprenticeship.^119^ His intentional alteration of the text of the pharmacopoeia for a new audience, an extension or ‘articulation’ of the original with additional material, made for a more articulate entity with improved usefulness. Similarly, Cole’s 1661 version might be construed as a further articulation of the same text, but equally a disarticulation as some linkages (of Culpeper’s) are dissolved for political purposes, underlining the need for continual reassessment in the dynamic of translating medical texts. Many insertions in Cole’s version also did not survive into the next edition of the translation. This history of translation in science has provided the analytical means to understand its sociocultural-political circumstances, as I have attempted to show here.

## Conclusions

The medical editors seem to have performed sufficiently what Cole commissioned them to do in 1661, by editing out Culpeper’s scurrilous comments and providing further uses and benefits for many of the compound remedies based on their physicians’ knowledge and experience. They further dismissed his criticisms of instructions for preparing some of the remedies to remove the insinuation of a poor knowledge of pharmacy among College physicians, and perhaps among themselves also, and played down his concerns about the safety of some formulations by insisting he had failed to fully appreciate their formulations. On the other hand, they kept Culpeper’s warnings to the untrained about meddling with some remedies for their new audience of physicians and showed a limited interest in discussing the chymical remedies.

For his part, Peter Cole showed himself to be a supporter of Galenic remedies and hostile to adepts of chymical medicines, whom he considered ‘strongly tainted with imposture’.^120^ This might explain why he tolerated the absence of commentary from both Culpeper and the medical editors in the section ‘chymical medicines more frequent in use’ across all editions of Culpeper‘s translation discussed here. Cole’s main concern in 1661 was maintaining his business, bringing his investments to bear fruit and avoiding the charge of sedition because of his publications during the Civil Wars. He must have perceived as a necessary expedient in changed times his sycophancy towards the College, his framing of the usefulness of the translation as limited to ships’ surgeons and to the poor who could not afford a doctor, and his redaction of the political and religious views of his most successful author Culpeper, which he too formerly professed.

The development of his translations of the London Pharmacopoeia shows Culpeper moving from the earnest dedication and measured criticism of the first edition—he only conceived the ambitious project of a ‘whole moddel of physick’ in English for his fellow countrymen and women after its success—to more assured but often strident attacks in 1653, piqued by his failing health and with no sign of the return of Christ to the world.^121^ Moreover, he was frustrated at having to translate a new edition of the pharmacopoeia which hid the authorship of remedies and still contained medicines no longer in use, written by doctors who seemingly made changes for the sake of change with little purpose and who corrected few of the errors he had picked out in his extensive commentary on the compound medicines of the 1618 Pharmacopoeia. These concerned the pharmaceutical preparation, the safety and the cost of various compound medicines. Culpeper also provided personal testimonies to the effectiveness of a few remedies. Culpeper comes over as a reformer in his rejection of the use of excrements and vomiting medicines, concern for public safety regarding medicines and understanding of what a pharmacopoeia should establish. Yet his preference for herbal simples over official medicines and his apparent lack of familiarity with true chymical medicines suggests a more traditional orientation. The bowdlerisation of Culpeper’s translation of the London pharmacopoeia in the 1661 edition seems not to have greatly dented his reputation, however, since the *Orders and Directions* for managing plague issued by the Mayor of London and his aldermen in 1665 named Culpeper on the title-page as one of the diverse physicians and doctors who had ‘set forth and approved’ recipes for the prevention and cure of that contagion. Moreover, after Cole’s death that same year, the version of Culpeper’s translation to be reprinted over the next fifty years was that of his 1653 work, only less redacted than the version edited by physicians.^122^

As for the Fellows and licentiates of the College of Physicians, they had not yet regained control over the publishing rights of their sacred text and anyway the second Latin edition did not evidence advances in science and thought at the College. Thanks to the redactions in Peter Cole’s version of the *Pharmacopoeia Londinensis*, the Restoration period saw no more printing of Culpeper’s scurrilous attacks on them, although his able critique of its contents was also rubbed out.

## Footnotes

1 Frederick N. L. Poynter, ‘Nicholas Culpeper and his Books’, *Journal of the History of Medicine and Allied Sciences*, XVII (1962), 152–67.

2 Dmitri Levitin, ‘“Made Up from Many Experimentall Notions”: The Society of Apothecaries, Medical Humanism, and the Rhetoric of Experience in 1630s London’, *Journal of the History of Medicine and Allied Sciences*, 70 (2015), 549–87 pointed out the high social origins and status of most apothecaries at that time, which should imply a decent command of Latin, although Culpeper had taught Latin to his first Master when an apprentice apothecary in the 1630s. The advantages of his translation to apothecaries listed by Culpeper did not include being an aid to understanding the text.

3 For the ‘humble retailing apothecary’ see Ray S. Roberts, ‘The Personnel and Practice of Medicine in Tudor and Stuart England: Part II London’, *Medical History*, 8 (1964), 217–34, 226.

4 Mary E. Fissell, ‘The Marketplace of Print’, in Mark S. R. Jenner and Patrick Wallis, eds, *Medicine and the Market in England and its Colonies c.1450-c.1850* (London: Palgrave Macmillan, 2007), 108–32. Fissell reported (p. 115) that Culpeper’s name ‘is associated with an astounding 8.5 per cent of all vernacular editions of medical books produced 1641–1740’.

5 Charles Webster, *The Great Instauration; Science, Medicine and Reform 1626–60* (London: Duckworth, 1975), 267–71.

6 Harold J. Cook, *The Decline of the Old Medical Regime in Stuart London* (Ithaca and London: Cornell University Press, 1986), 121.

7
*Mercurius Pragmaticus*, pt. Ii, no. 21 (4–9 Sept 1649), cited in J.B. *A faire in Spittle Fields* (London, 1652). ‘Failed apothecary’ refers to Culpeper’s abandonment of his apothecary training in 1640 before he had completed his 7 years. Mary Rhinelander McCarl, ‘Publishing the Works of Nicholas Culpeper, Astrological Herbalist and Translator of Latin Medical Works in Seventeenth-Century London’, *Canadian Bulletin of Medical History*, 13 (1996), 225–76 and Clare Fowler, *Pharmacopoeia Londinensis 1618 and its Descendants (*London: Royal College of Physicians, 2018), 87–116 cite this attack, the latter noting that no reference to the publication was to be found in the College’s *Annals* and the subsequent attack in print by William Johnson, the ‘College’s chemist’ on this ‘obscene book’ was not backed by the College (p. 109).

8 Fowler, *Pharmacopoeia Londinensis*, 103; Peter Cole, *King Charles his Tryal at the High Court of Justice Sitting in Westminster Hall* (London: Printed by J.M. for Peter Cole, Francis Tyton and John Playford, 1650).

9 Christopher Hill, *Change and Continuity in Seventeenth-century England* (London: Weidenfeld and Nicolson, 1974), 171.

10 Harold J. Cook, ‘Policing the Health of London: The College of Physicians and the Early Stewart Monarchy’, *Social History of Medicine*, 2 (1989), 1–33, but see Levitin ‘Experimentall Notions’, 578–80, where he argued that the College of Physicians had considered imposing its control over apothecaries by means of a centrally organised pharmacopoeia as early as 1585 in advance of the politics of Stuart absolutism.

11 William Coles, *The Art of Simpling* (London: Printed for Nathaniel Brook, 1656), 76–78; Matthew Mackaile, *Moffet-Well; Or, a Topographico-Spagyricall Description of the Mineral Wells at Moffet…As, also at Oyly-Well…to these is subjoined A Character of Mr Culpeper and his Writings* (Edinburgh: Printed for Robert Brown, 1664), 145–96.

12 Only one example of Culpeper’s ‘decumbiture’ horoscopes, cast by him for the time his patient fell ill, made it into print, with the comment attached: ‘I have many decumbitures by me; But I want time to insert them, or if I did not, I would not blot paper with them. Be pleased to accept of this one in lieu of all the rest.’, Nicholas Culpeper, *Semeiotica Uranica; Or, An Astrological Judgment of Diseases from the Decumbiture of the Sick* (London: Nathaniel Brooke, 1651), 58–69, 58. In an earlier note he claimed to have treated more than 20 cases of intermitting tertian fever in ‘Febrilia, Or treatise of fevers in general’ dated 7 February 1645/6 in Nicholas Culpeper, *Culpeper’s Last Legacy* (London: Nathaniel Brooke, 1655), 15.

13 Culpeper, *Pharmacopoeia Londinensis* (London: Peter Cole, 1653), 58, where he observed that physicians ‘cannot write true Latin’ and tended to abbreviate their words by omitting the last letters of a word, thus disguising the word’s gender and case and often its meaning. He also resolved a Latin misprint in his source text to make sense of the instructions for making pills of Rudius. Nicholas Culpeper, *A Physical Directory* (London: Peter Cole, 1650), 64.

14 Robert L. Martensen, ‘Habit of Reason: Anatomy and Anglicanism in Restoration England’, *Bulletin of the History of Medicine*, 66 (1992), 511–35; Michael Heyd, ‘Medical Discourse in Religious Controversy: The Case of the Critique of “Enthusiasm” on the Eve of the Enlightenment’, *Science in Context*, 8 (1995), 133–57.

15 Elizabeth Lane Furdell, ‘“Reported to be Distracted”: The Suicide of Puritan Entrepreneur Peter Cole’, *The Historian*, 66 (2004), 772–92.

16 McCarl, ‘Publishing*’* 273–76, matched these hybrid versions of Culpeper’s translation of the pharmacopoeia to Cole’s 1661 version in her list of editions of Culpeper to 1700 rather than to a redacted version of Culpeper’s 1653 translation.

17 George Urdang, *Pharmacopoeia Londinensis of 1618* (Madison: State Historical Society of Wisconsin, 1944).

18 Fowler, *Pharmacopoeia Londinensis*, 106.

19 Benjamin Woolley, *The Herbalist: Nicholas Culpeper and the Fight for Medical Freedom* (London: HarperCollins Publishers, 2004), 139–42; Olav Thulesius, *Nicholas Culpeper English Physician and Astrologer* (Basingstoke: The Macmillan Press, 1992); Graeme Tobyn, *Culpeper’s Medicine: A Practice of Western Holistic Medicine* (Shaftesbury: Element Books, 1997).

20 Michael Walkden, ‘“That they may vomit out their folly”: The gut-mind axis and hellebore in early modern England’, *Journal of British Studies*, 61 (2022), 535–62.

21 I have provided more examples of Culpeper’s points of criticism and the responses of the medical editors in a related article: Graeme Tobyn, ‘Culpeper Versus Physicians: Deficiencies in the London Pharmacopoeia, his Commentary and their Responses’, *Pharmaceutical Historian*, 55 (2025), 129–45.

22 Charles Webster, ‘The College of Physicians: “Solomon’s House” in Commonwealth England’, *Bulletin of the History of Medicine*, 41 (1967), 393–412, 395.

23 Culpeper, *A Physicall Directory* (London: Peter Cole, 1649), ‘The Colledge to the candid reader’, B2r-B2v. Only on one page had the College given an inkling of therapeutic use of simples, when they listed such groupings as the five *opening* roots, the four *cordial* flowers and the four greater *hot* seeds (my italics) as ‘An interpretation of certain common names’. Culpeper, A *Physicall Directory*, 79.

24 Fowler, *Pharmacopoeia Londinensis,* 106.

25 Robert Burton, *The Anatomy of Melancholy* (1621), cited in Andrew Wear, *Knowledge and Practice in English Medicine 1550–1680* (Cambridge: Cambridge University Press, 2000), 93. Patrick Wallis, ‘Apothecaries and the Consumption and Retailing of Medicines in Early Modern London’, in Louise Hill Curth, ed, *From Physick to Pharmacology: Five Hundred Years of British Drug Retailing* (Aldershot: Ashgate, 2006), 13–27, analysed the apparently complete inventory records of eight London apothecary shops in the 1660s and 1670s showing that three kept 40 or 50 different commodities in their shops, three had over 100 and two over 200.

26 Cited in McCarl, ‘Publishing*’,* 232.

27 Culpeper, A *Physicall Directory*, 1649, 78.

28 Culpeper, *Pharmacopoeia,* 1653, 162. In the seventeenth-century Momus was seen as a comic god of blame.

29 Culpeper, A *Physicall Directory,* 1649, B3v.

30 Culpeper, A *Physicall Directory*, 1649, A1v-A3r; Webster, *Great Instauration*, 533–34.

31 Culpeper, A *Physicall Directory,* 1649, A1v-A3r, B1v.

32 Culpeper, A *Physical directory* (London: Peter Cole, 1650), 182, 201.

33 Culpeper, A *Physical Directory*, 1650, B2r.

34 Culpeper, *A Physical Directory* (London: Peter Cole, 1651), 138.

35 Culpeper, A *Physical Directory,* 1651, *The epistle dedicatory,* A2r.

36 Culpeper, *Pharmacopoeia Londinensis*, 1653, 147, 118.

37 Webster, ‘Solomon’s House’, 40; Cook, *Decline*, 112. Sir George Clark, *A history of the Royal College of Physicians of London*, 2 vols (Oxford: Clarendon Press, 1964), I, 278, suggests some changes were ‘impractical’ and the College became ‘more oligarchical and exclusive’ as a result.

38 Clark, *History*, I, 286–87; Fowler, *Pharmacopoeia*, 84.

39 McCarl, ‘Publishing’, 233; John Barnard, ‘London Publishing 1640–1660: Crisis, Continuity and Innovation’, *Book History*, 4 (2001), 1–16.

40 Clark, *History*, I, 286–87, 345.

41 George Urdang ‘The Development of Pharmacopoeias’, *Food, Drug, Cosmetic Law Journal*, 8 (1953), 69–91.

42 Urdang, ‘Development of Pharmacopoeias’, 75; Urdang, *Pharmacopoeia*, 25; Culpeper, *Pharmacopoeia*, 1653, 66.

43 Culpeper, *Pharmacopoeia,*1653, 66.

44 Culpeper, *Pharmacopoeia*, 1653, 117, 102. *Pharmacopoeia Augustana iussu et auctoritate amplissimi senatus a Collegio medico rursus recognita, ac elaboratior et auctior, nunc sextum in lucem emissa* (Augusta Vindelicorum, 1613) 6, 120–21, https://babel.hathitrust.org/cgi/pt?id=ucm.5327360644&view=1up&seq=390. Fowler, *Pharmacopoeia*, 67–70. Urdang, *Pharmacopoeia*, 63–73, 71, reviewed the sources of the London Pharmacopoeia and identified only 10 recipes taken from the *Pharmacopoeia Augustana*.

45 Culpeper, *Pharmacopoeia,* 1653, 35–36; Culpeper, A *Physicall Directory,* 1649, 163, 279.

46 Nicholas Culpeper, *The English Physitian* (London: Peter Cole, 1652), A2v.

47 Urdang, *Pharmacopoeia*, 8.

48 Valentina Pugliano, ‘Pharmacy, Testing, and the Language of Truth in Renaissance Italy’, *Bulletin of the History of Medicine*, 91 (2017), 233–73.

49 Ibid., 240.

50 Ibid., 272.

51 Culpeper *Pharmacopoeia*, 1653, 34.

52 Culpeper, *Pharmacopoeia*, 1653, 34. This phrase was singled out for inclusion on the title page of a pirated edition of Culpeper’s translation of the pharmacopoeia in 1654 and was excised by the doctors in Cole’s 1661 edition. Elaine Leong, ‘“Herbals She Peruseth”: Reading Medicine in Early Modern England’, *Renaissance Studies*, 28 (2014), 556–78, illustrates how Culpeper’s herbal was used critically and selectively by one reader for trying cures, planting herbs and making medicines and provides an idea of how the expanded sections on simples in Culpeper’s translation of the pharmacopoeia was intended to provide similar benefit.

53 Culpeper, *Pharmacopoeia,* 1653, 70; Culpeper, A *Physicall Directory*, 1649, 108. The medical editors of the 1661 version had Culpeper calling these *fine* cooling drinks in the heat of summer and added other benefits of their own. Culpeper, *Pharmacopoeia,* 1661, 121.

54 Culpeper, *Pharmacopoeia*, 1653, 124.

55 Culpeper *Pharmacopoeia*, 1653, 34.

56 Graeme Tobyn, ‘Did the Astrological Content of Culpeper’s *English Physitian* Undermine its Usefulness?’, *Culture and Cosmos*, 26 (2022), 35–53.

57 Culpeper, *A Physicall Directory*, 1649, 76; Culpeper, *Pharmacopoeia*, 1653, 53.

58 Culpeper, *Pharmacopoeia*, 1653, 31–32; Caroline Petit, ‘Galen, Pharmacology and the Boundaries of Medicine: A Reassessment’, in Lemmart Lehnhaus and Matteo Martelli, eds, *Collecting Recipes: Byzantine and Jewish Pharmacology in Dialogue* (Berlin, Boston: De Gruyter, 2017), 51–79; Culpeper*, Pharmacopoeia*, 1653, 52.

59 Culpeper, *Pharmacopoeia*, 1653, 53.

60 Glenn Sonnedecker, ‘Have you heard’, *Pharmacy in History*, 71, pointed out that the May 1618 edition had no drekapotheke among the simples and Urdang, *Pharmacopoeia*, 80–81, noted that none of the urines described among the simples had found their way into the 1618 formulas for compounded medicines, but a plaster for the head containing dove’s dung seems to have been approved (*Pharmacopoeia Londinensis,* May 1618, 143; December 1618, 167).

61 Culpeper, *Pharmacopoeia*, 1653, 52. Urdang, *Pharmacopoeia*, 40, 44–45.

62 Culpeper, *Pharmacopoeia*, 1653, 58–61. Leonardo Fioravanti, *Three Exact Pieces* (London: G. Dawson, 1652), Preface by Johnson addressed to ‘Friend Culpeper’.

63 Culpeper, *Pharmacopoeia,* 1653, 63; Culpeper, *A Physicall Directory*, 86. The medical editors of Cole‘s 1661 version dismissed the concern by excising these comments and adding further indications of their own for the angelica water.

64
*The British Pharmacopoeia 1914* (London: Constable & Co. Ltd., 1914), 293.

65 J. Erik LaPort, ‘Elixir Proprietatis’. Research.edu  https://www.academia.edu/25948048/Elixir_Proprietatis_The_Property_of_Humanity; Aleksander K. Smakosz, ‘Pharmacopoeial Elixirs (of life) (17th–19th Century)’, *Herbal History Research Network,*  https://www.herbalhistory.org/home/pharmacopoeial-elixirs-of-life-17th-19th-century/.

66 Culpeper, *A Physical Directory*, 1650, ‘To the impartial reader’.

67 Culpeper, *Pharmacopoeia*, 1653, 151.

68 Culpeper, *Pharmacopoeia,* 1653, 127, 110, 63.

69 ‘Contusis contunendis infundatur per horas viginti quattuor in alembico vitreo super cinerem calidos deinde distillentur ut artis est’ are the instructions for preparing *Aqua imperialis* translated by Culpeper as ‘the things to be bruised, being bruised, let them be infused twenty four hours in a glass still over hot ashes and then distilled according to art’. *Pharmacopoeia Londinensis: in qua medicamenta antiqua et noua vsitatissima, sedulò collecta, accuratissime examinata, quotidianâ experientia confirmata describuntur. Diligenter reuisa, denuo recusa, emendatior auctior. Opera Medicorum Collegij Londinensis. Ex serenissimi Regis mandato cum R.M. priuilegio* (Royal College of Physicians of London, 1618), 10.

70 Culpeper, *A Physicall Directory*, 88–89; Culpeper, *Pharmacopoeia*, 1653, 66. The medical editors retained Culpeper‘s comment on distilling in a water bath in Cole’s 1661 version but it no longer had a clear meaning in relation to the redacted text.

71
*Pharmacopoeia Londinensis*, 1618, 57.

72 Culpeper, *A Physicall Directory*, 1649, 147.

73
*Pharmacopœia Londinensis collegarum: hodie viventium studiis ac symbolis ornatior* (Royal College of Physicians of London, 1650), 87; Culpeper, *Pharmacopoeia*, 1653, 120. The order of making sugar of roses in the 1618 Pharmacopoeia ‘in umbra siccentur et terantur postea optime in mortario lapideo tunc cum unciis duodecim sacchari optimi soluti in aqua rosarum rubrarum, coquantur secundum artem, dum aqua fuerit absumpta’ was changed in the second Latin edition so that the powedered rose flowers were added only at the end to preserve the colour: ‘Fundatur saccharum ad ignem in aqua rosar. Rubrarum & succi earundem ana unciis duabus. His sensim absumptis, pulveratas rosas admisce’.

74 Culpeper, *Pharmacopoeia*, 1653, 124, 121, 158.

75 Culpeper, *Pharmacopoeia*, 1653, 134. The medical editors of the 1661 text removed Culpeper’s comments on the powders and electuary adding uses of their own, including the making of lozenges out of the electuary for children with worms.

76 Urdang, *Pharmacopoeia,* 8, pointed out that modern pharmaceutical literature classifies aromatic waters, tinctures and extracts with galenicals, even though they were unknown to Galen, and that the argument between Paracelsians and Galenists was thought to reside in the preparation of inorganic chemicals for internal medication.

77 Fowler, *Pharmacopoeia*, 54–56, 74. Webster, ‘Solomon’s House’, 408.

78 Levitin, *Experimentall Notions*, 568–69.

79 Cook, *Decline*, 112–13, 117. Culpeper, *Pharmacopoiea*, 1653, 53, acknowledged that an alchemist now operated in the College garden, but had complained in 1651 that the gate was always closed. The term ‘chymical’ is used by some historians to refer to seventeenth-century theory and practice not adequately communicated by the contrasting meanings of the terms chemistry and alchemy. William R. Newman and Laurence M. Principe, ‘Alchemy vs. Chemistry: The Etymological Origins of a Historiographic Mistake’, *Early Science and Medicine*, 3 (1998), 32–65.

80 This was the only edition of this version of Culpeper’s translation of the *Pharmacopoeia*, although I have found another edition of it, dated 1665 and not listed in the British Library catalogue, in the United States National Library of Medicine http://resource.nlm.nih.gov/2751057R.

81 Culpeper, *Pharmacopoeia*, 1661, 157.

82 Culpeper, *Pharmacopoeia*, 1661, ‘the printer to the reader’.

83 Ibid.

84 Culpeper, *Pharmacopoeia,* 1661, 169–70.

85 Hill, *Change and Continuity*, 168.

86 Culpeper, *Pharmacopoeia,* 1661, ‘The Printer to the Reader’.

87 Cook, *Decline,* 133–82.

88 Adrian Johns, *The Nature of the Book* (Chicago: University of Chicago Press, 1988), 228; McCarl, ‘Publishing’, 269.

89 J. Walker, ‘The Censorship of the Press During the Reign of Charles II’, *History* 35 (1950), 223–27.

90 Furdell, ‘Reported to be Distracted’, 772–92.

91
*Pharmacopoeia Londinensis* (1661), B1r-B1v.

92 Marttila, Ville, ‘New arguments for New Audiences: A Corpus-based Analysis of Interpersonal Strategies in Early Modern English Medical Recipes’, in Irma Taavitsainen and Päivi Pahta, eds, *Medical Writing in Early Modern English* (Cambridge: CUP, 2011), 135–57; Peter Murray Jones, ‘Book Ownership and the Lay Culture of Medicine in Tudor Cambridge’, in Hilary Marland and Margaret Pelling, eds, *The Task of Healing: Medicine, Religion and Gender in England and the Netherlands (1485–1800)* (Rotterdam: Erasmus Publishing, 1996), 49–68.

93 Culpeper, *Pharmacopoeia*, 1661, 153.

94 62% based on 368 entries among 593 recipes in the 1653 text. Note that the layout of simple oils by infusion and decoction differs in the 1661 version.

95 Culpeper, *Pharmacopoeia,* 1661, ‘The Printer to the Reader’.

96 Culpeper, *Pharmacopoeia*, 1661, 170.

97 A.C. is very unlikely to be either Abel Collier or Abner Coe who were College physicians active around 1660 or, casting the net more widely to include those extra-licentiate physicians whom the College made honorary fellows in 1664, none had the initials A.C. William Monk, *The Rolls of the Royal College of Physicians of London* (Published by the College, Pall Mall East, London, 1878); John Venn and J. A. Venn, *Alumni Cantabrigienses* (Cambridge: CUP, 1922). Retrieved from: https://archive.org/details/alumnicantabrigipt1vol1univiala/page/n5; *Athenae Oxonienses* (London: Tho. Bennet, 1691); Clark, *History,* 1, 304–307, (fellows), 312–17 (honorary fellows); *Annals of the College of Physicians*, *V*ol.4 f.87a – f.87b; E. Ashworth Underwood, Cecil Wall and Hector Charles Cameron, *A History of the Worshipful Society of Apothecaries of London*, Vol. 1 (London: Oxford University Press, 1963), 353.

98 Pharmacopoeia Londinensis (1661), Preface *The Printer to the Reader*, B1v.

99 Oxford Dictionary of National Biography, ‘Abdiah Cole’.

100 John Schroder, *The Compleat Chymical Dispensatory* (London: printed by John Darby for Richard Chiswell and Robert Clavell, 1669), The Epistle, A1r-A1v; Michelle Pfeffer, ‘The Society of Astrologers (c.1647–1684): Sermons, Feasts and the Resuscitation of Astrology in Seventeenth-century London’, *The British Journal for the History of Science*, 54 (2021), 133–53. The publisher of Culpeper’s astrological works was Nathaniel Brooks. For Cole’s unpicking of some of the astrological structure of Culpeper’s herbal after the author’s death, see Tobyn, ‘Astrological content’, 45.

101 Charles Webster, *William Rand*, Oxford Dictionary of National Biography; Webster, *Great Instauration*, 305, 533–34. W.R. is unlikely to be William Ramesey MD, author of *Astrologia Restaurata* (1653) who was hostile to all but the university-trained physician and may not have supported the translation of the pharmacopoeia.

102 Culpeper, *Pharmacopoeia*, 1661, 130.

103 Culpeper, *Pharmacopoeia*, 1661, 125.

104 Culpeper, *Pharmacopoeia*, 1653, 168. Wood sorrel, Parkinson wrote, was ‘called by the apothecaries in their shoppes Alleluja…because about that time it is in flower Alleluja in antient times was wont to bee sung in the churches’, thus Culpeper’s pun. John Parkinson, *Theatrum botanicum* (London: Thomas Cotes, 1640), 747.

105 Culpeper, *Pharmacopoeia*, 1661, 189–90.

106 Culpeper, *Pharmacopoeia*, 1653, 176.

107 Culpeper, *Pharmacopoeia*, 1661, 199. This slight on the religion of College Fellows reappeared in the 1669 edition of Culpeper’s translation of the pharmacopoeia published by John Streater, and subsequently. Similarly reinstated was Culpeper’s attack on the skills of College members in alchemy, in the entry on chymical oil of wormwood.

108 Culpeper, *Pharmacopoeia*, 1653, 64.

109 Culpeper, *Pharmacopoeia*, 1661, 103. This recipe appeared without any comments in subsequent editions of Culpeper’s translations.

110 Culpeper, *Pharmacopoeia*, 1653, 167; Culpeper, *Pharmacopoeia*, 1661, 188.

111 Entries for 25 out of 36 spirits and compound waters and 23 of the 28 honeys, oxymels and robs.

112 Forty-two out of 59 oils, 43 out of 57 ointments, all 3 cerecloths and 32 out of 54 plasters, totalling 120 entries among 173 recipes.

113 Culpeper, *Pharmacopoeia*, 1661, 104.

114 Culpeper, *Pharmacopoeia*, 1661, 186.

115 Culpeper, *A Physicall Directory*, 83.

116 Allen G. Debus, *The English Paracelsians* (New York: Franklin Watts, Inc., 1966), 49–85.

117 Culpeper, *Pharmacopoeia,* 1661, 71; cf. Culpeper, *English Physitian*, 7–8.

118 Culpeper, *Pharmacopoeia,* 1661, 111.

119 Tara Alberts et al., ‘Translating Medicine, ca. 800–1900: Articulations and Disarticulations’, *Osiris,* 37 (2022), 1–23.

120 Culpeper, *Pharmacopoeia*, 1661, ‘the printer to the reader’.

121 Mary E. Fissell, *Vernacular Bodies: The Politics of Reproduction in Early Modern England* (Oxford: Oxford University Press, 2006), 135–56, 142.

122
*The Orders and Directions of the Right Honorable the Lord Mayor and Court of Aldermen, to be Diligently Kept and Observed by the Citizens of London during the Time of the Present Visitation of the Plague* (London: Printed for George Horton, 1665).

